# Maternal-fetal attachment and anxiety in pregnant women who conceived through assisted reproductive technology: A longitudinal study

**DOI:** 10.18502/ijrm.v19i12.10058

**Published:** 2022-01-12

**Authors:** Fahimeh Ranjbar, J. Catja Warmelink2comma 3comma, Robab Mousavi, Maryam Gharacheh

**Affiliations:** ^1^Nursing Care Research Center, Iran University of Medical Sciences, Tehran, Iran.; ^2^Department of Midwifery Science, Amsterdam Public Health Research Institute, VU University Medical Center, Amsterdam, the Netherlands.; ^3^Department of General Practice and Elderly Medicine, University Medical Centre Groningen, University of Groningen, Groningen, the Netherlands.; ^4^Midwifery Academy, Academie Verloskunde Amsterdam Groningen (AVAG), the Netherlands.; ^5^School of Nursing and Midwifery, Iran University of Medical Sciences, Tehran, Iran.

**Keywords:** Attachment, Maternal fetal relations, Assisted reproductive technology, Pregnancy, Anxiety.

## Abstract

**Background:**

Pregnancy through assisted reproductive technology (ART) is a stressful experience that may affect prenatal attachment. However, maternal-fetal attachment (MFA) and anxiety in pregnancy after ART are understudied in Iran.

**Objective:**

To compare changes in MFA and pregnancy-related anxiety (PRA) in the first and third trimester of pregnancy in women who conceived through ART compared to those who conceived naturally.

**Materials and Methods:**

This longitudinal study was conducted in 2019 with 187 pregnant women (ART conception = 43, natural conception = 144). Participants were recruited using the consecutive sampling method from a prenatal clinic in Tehran. The Cranley MFA Scale and the Van Den Bergh PRA Questionnaire were used to collect the data.

**Results:**

The MFA score in the 12 ${}^{\text{th}}$ wk of gestation was lower in the women who conceived with ART compared to in the women who conceived naturally, but there was no statistically significant difference between the groups in wk 36. MFA in both groups was significantly higher at gestational wk 36 than wk 12 (p ≤ 0.001). The increase in MFA score was significantly higher in the women who conceived with ART than in those who conceived naturally (p ≤ 0.001). The anxiety score declined in the two groups and no statistically significant difference was observed in the changes of anxiety scores between the two groups (p = 0.84).

**Conclusion:**

Pregnant women who conceived through ART were as attached to their fetus in the third trimester of pregnancy as other women and did not experience more PRA.

## 1. Introduction

Undergoing assisted reproductive technology (ART) can be a stressful experience for couples (1). Pregnant women with infertility treatment face more psychological and mental health problems (2), and more medical complications in pregnancy and childbirth (3). Thus, pregnancy, which is supposed to be a pleasant time, can turn into a challenging period for infertile couples (2). Studies over two decades have shown that compared with those who conceive naturally, women who conceive through ART report comparable symptoms of general anxiety (4, 5). However, looking at pregnancy-related anxiety (PRA), some studies have shown that women who conceive through ART experience a higher level of PRA than women who conceive naturally (5, 6). The increased PRA does not seem to negatively affect maternal-fetal attachment (MFA) in women who conceived by ART but the findings on PRA and MFA can be contradictory (7).

Maternal attachment is a major component of maternal identity that is important in adapting to motherhood. It is well recognized that the quality of the mother-fetus relationship is related to the quality of the mother-infant relationship after childbirth, and is the main factor affecting the child's cognitive and emotional development (8). This attachment has also been known to link to pregnancy-related health practices, such as receiving prenatal care and reducing alcohol consumption during pregnancy (9). Therefore, it is essential to identify the factors influencing MFA due to the impacts on the future child's development and the mother and child health outcomes (8).

A recent review showed that in women who became pregnant following ART, the level of MFA was either similar to or higher than the MFA in women without ART, regardless of pregnancy stage (7). The desired pregnancy is associated with positive emotions and feelings experienced by a mother towards her child (10) and as pregnancy following ART appears for most previously infertile women to be a much-desired condition, attachment with such a desired fetus is likely to be stronger (11). However, this pleasant event may be accompanied by psychological changes leading to stress and anxiety in these women (12). A study showed that in the third trimester of pregnancy, women who conceived through ART reported lower state and trait anxiety, but higher PRA than naturally pregnant women (6). According to a systematic review, in ART pregnant women, PRA, and specific anxiety about the survival of their child and their parenting abilities increased compared to non-ART women (5). However, none of the above-mentioned studies and reviews on MFA and PRA in pregnant women have been conducted in Islamic Middle East regions, even though the Middle East hosts one of the strongest ART industries in the world (13).

The objective of the study was to examine MFA and PRA in the first and last trimester in pregnant women who conceived through ART compared to those who conceived naturally in Iran. The findings of the study can broaden the understanding of MFA and PRA in pregnant women. Furthermore, they can help healthcare professionals to identify pregnant women at risk for altered MFA or PRA and to provide appropriate care for these women.

## 2. Materials and Methods 

This longitudinal study was conducted to compare changes in MFA and PRA in the first and third trimester of pregnancy in pregnant women who conceived through ART vs. those who conceived naturally, from March to October 2019.

Considering the unequal distribution of these two populations, to determine the study sample size at 95% confidence level and 80% power, the sample size formula for unequal sized groups was used (given the ratio of 3 to 1). Accordingly, a consecutive sample of 187 pregnant women (ART conception = 43, natural conception = 144) was selected from the prenatal clinic of Shahid Akbarabadi Hospital, Iran.

The women who met the following inclusion criteria were included in the study: married women of Iranian nationality, aged between 18 and 45 yr, and becoming pregnant either spontaneously or through ART (including through in vitro fertilization, intracytoplasmic sperm injection). Infertile women who become pregnant through ovulation induction, donated gametes or embryos, or intrauterine insemination, and those who developed pregnancy complications during the study (such as hemorrhage, gestational hypertension, gestational diabetes, etc.), or had mental health disorders (such as depression) were excluded.

The participants were requested to complete the self-administered questionnaires in gestational wk 12 and 36. In the group of pregnant women who conceived naturally, six women were excluded (three for preterm labor, two for intra-uterine fetal death, one for abortion), and in the ART group, seven women were excluded (two for preterm labor, five for abortion).

A socio-demographic form, the Cranley MFA Scale (MFAS) and the Van Den Bergh PRA Questionnaire (PRAQ-17) were used to collect the data. MFAS included 24 items under five domains which included role-taking (four items), differentiation of self from the fetus (four items), interaction with the fetus (five items), attributing characteristics to the fetus (six items), and giving of self (five items). The items each comprised potential responses of 1 (Definitely no), 2 (No), 3 (Uncertain), 4 (Yes), and 5 (Definitely yes). The total score ranged from 24 to 120. The Cronbach's alpha coefficient of the instrument was 0.85 (14). Validity for the Persian version of the instrument was assessed by experts in the field of obstetrics, psychology, and reproductive health, and its reliability was confirmed with the Cronbach's alpha coefficient of 0.83 (15).

PRAQ-17, which was used to examine PRA, contained 17 items with five factors: fear of childbirth (three items), fear of giving birth to a child with physical or mental health problems (four items), fear of the marital relationship change (four items), fear of changes in mood and its impacts on the child (three items), and self-centered fear or fear of the personal life changes (three items). Each item was ranked between one and seven. The total score ranged from 17 to 119 (16). Validity and reliability (Cronbach's alpha = 0.78) of the Persian version of PRAQ-17 was confirmed by Askarizadeh et al. (17).

### Ethical considerations

The Ethics Committee of Iran University of Medical Sciences approved the study (Code: IR.IUMS.REC.1397.1026). A written consent form was signed by all the participants and they were advised of their right to withdraw from the research at any time.

### Statistical analysis

Descriptive statistics (such as means, standard deviations, and frequencies) were applied to summarize the data. The Chi-square test was used to analyze categorical variables, and independent samples student's *t* test, paired *t* test and Pearson correlation test were used to assess the association between independent variables and the dependent variable. Data were analyzed through the Statistical Package for the Social Sciences (SPSS) statistical software version 16 (IBM Corp., Armonk, NY, USA) and p < 0.05 was considered statistically significant.

## 3. Results

Of 200 women invited, 187 were included in the study. The mean age of women who conceived through ART and naturally was 33.11 ± 4.39 and 31.81 ± 6.13 yr, respectively. 41.9% of women who conceived by ART and 42.4% of women who conceived naturally had a secondary school education. Participant characteristics at wk 12 are summarized in table I, which showed that the characteristics of the two groups were not significantly different.

As shown in table II, results of the paired *t* test indicated that at the 12 ${}^{\text{th}}$ wk of gestation, the MFA score for the women who conceived by ART was lower compared to women who conceived naturally (ART = 87.67; NC = 92.55; p < 0.01), but that MFA in both groups was significantly higher at gestational wk 36 than at wk 12, and the increase in MFA score was significantly higher in the women who conceived through ART than in those who conceived naturally. Compared to women who conceived naturally, the scores of women who conceived by ART were higher at wk 12 for the MFAS sub-scales “role taking” (ART = 12.64; NC = 5.26; p ≤ 0.001) and “differentiating of self from fetus” (ART = 5.75; NC = 9.76; p ≤ 0.001); and at wk 36, women who conceived by ART had significantly lower scores than women who conceived naturally for the MFAS sub-scale “attributing characteristics to the fetus” (ART = 0.37; NC = 6.39; p ≤ 0.001). PRA was not significantly different between the two groups. The PRA score declined in both groups and no statistically significant difference was observed in changes of PRA scores between the two groups.

There was a significant negative relationship between MFA and PRA at the 12 ${}^{\text{th}}$ wk of gestation in the ART-conception women (p = 0.02) but no statistically significant correlation was found between MFA and PRA at the 36 ${}^{\text{th}}$ wk of gestation in either ART- or natural-conception women (Table III).

**Table 1:** Characteristics of participants


**Characteristics**	**ART-conception (n = 43)**	**Natural-conception (n = 144)**	**p-value**
** Age (yr)**			
** < 25**	2 (4.7)	23 (16.0)	0.19*
**25-29**	5 (11.6)	23 (16.0)	
**30-34**	19 (44.2)	40 (27.7)	
** ≥ 35**	17 (39.5)	58 (40.3)	
**Mean ± SD**	33.11 ± 4.39	31.81 ± 6.13	
** Education level**			
**Illiterate**	1 (2.3)	4 (2.7)	0.32**
**Less than secondary school**	8 (18.6)	43 (29.9)		0.50**
**Secondary school**	18 (41.9)	61 (42.4)		
**University**	16 (37.2)	36 (25)		
** Employment status**				
**Employed**	4 (9.3)	9 (6.3)
**Unemployed**	39 (90.7)	135 (93.7)
** Household income**			
**Low**	33 (76.7)	124 (86.1)	0.28**
**Middle**	9 (20.9)	18 (12.5)	
**High**	1 (2.4)	2 (1.4)	
** Parity**			
**0**	21 (48.8)	42 (29.2)	
**1**	13 (30.2)	49 (34.0)	0.08 ${}^{\#}$
**2**	7 (16.3)	38 (26.4)	
** ≥ 3**	2 (4.7)	15 (10.4)	
** Abortion**			
**0**	29 (67.4)	97 (67.4)	0.12**
**1**	8 (18.6)	33 (22.9)	
**2**	2 (4.62)	12 (8.3)	
** ≥ 3**	4 (9.3)	2 (1.4)	
Data presented as n (%). ${}^{*}$ Independent *t* test, ${}^{**}$ Fisher's exact test, ${}^{\#}$ Chi-square test, ART: Assisted reproductive technology			

**Table 2:** Mean scores of MFA and anxiety for the ART-conception group and natural-conception group


**Variables **	**12 ${}^{\text{th}}$ wk of gestation**	**36 ${}^{\text{th}}$ wk of gestation**	*p-value	** ${}^{\#}$ Difference**
** MFA**				
**ART-conception **	87.67 ± 10.45	102.9 ± 7.45	≤ 0.001	14.41 (10.44)
**Natural-conception **	92.55 ± 9.59	100.93 ± 8.02	≤ 0.001	8.37 (9.20)
**p-value	≤ 0.001	0.40	≤ 0.001
** Anxiety**				
**ART-conception **	51.9 ± 18.2	33.67 ± 11.68	≤ 0.001	-18.23 (14.12)
**Natural-conception **	51.36 ± 9.59	32.52 ± 13.36	≤ 0.001	-18.84 (18.49)
**p-value	0.87	0.61	0.84
Data presented as Mean ± SD. ${}^{*}$ Paired *t* test, ${}^{**}$ Independent *t* test, ART: Assisted reproductive technology, MFA: Maternal-fetal attachment, ${}^{\#}$ The difference between 36 ${}^{\text{th}}$ wk of gestation and 12 ${}^{\text{th}}$ wk of gestation				

**Table 3:** Correlation between MFA and anxiety in each group at 12 ${}^{\text{th}}$ and 36 ${}^{\text{th}}$ wk of gestation


**Group**	**Wk of gestation**	
	**12 ${}^{\text{th}}$ wk**	**36 ${}^{\text{th}}$ wk**
**ART-conception **	r = -0.357	r = -0.079
	p = 0.01*	p = 0.61*
**Natural-conception **	r = -0.166	r = -0.101
	p = 0.20*	p = 0.22*
ART: Assisted reproductive technology, MFA: Maternal-fetal attachment, *Pearson correlation		

## 4. Discussion

The aim of the study was to compare MFA and PRA between pregnant women who conceived through ART and those who conceived naturally at gestational wk 12 and 36. The study's findings revealed that compared to women who conceived naturally, ART women had greater PRA at the 12 ${}^{\text{th}}$ wk of pregnancy. We hypothesized that stress caused by diagnostic and therapeutic evaluations of infertility, a history of treatment failures, or fear of losing the pregnancy may increase PRA and affect MFA.

### MFA

Our results showed that the mode of conception was related to MFA at wk 12 of gestation but there was no significant difference between the two groups at gestational wk 36. At the 12 ${}^{\text{th}}$ wk of gestation, the MFA score in women who conceived by ART was lower compared to in those who conceived naturally. These findings confirm the growing body of literature that increasing medical intervention in reproduction may have unintentional consequences for developing a confident attachment to the fetus (18). One study found a significant increase in attachment from first trimester to second trimester in women underging ART. After the first trimester screening, there was a significant increase in the parental-fetal attachment (19). Delay in the attachment at gestational wk 12 in pregnant women who conceived by ART may be explained by fear of pregnancy loss and uncertainty about the results of first trimester screening tests. Furthermore, a history of treatment failure or prior miscarriages may make pregnancy and the process of prenatal attachment more difficult (20). It has been indicated that in cases of infertility treatment failure, fatigue affects women's self-esteem and body image and causes them to be concerned about miscarriage, fetal malformations, and death (21). Whether the cause of infertility is a male factor or a female one, women directly experience pregnancy and hold themselves responsible for its consequences, and even if ART treatment is successful, they may not be able to easily overcome the negative feelings associated with infertility (22). In mothers who conceived by ART, pregnancy and childbirth are considered less of a continuum process, and more of a series of events (23). Therefore, satisfactory results of ultrasound examinations and screening in the first trimester can improve the mother's confidence and her attachment to the fetus.

In the present study, the rate of attachment changes in women who conceived through ART was higher than in those who conceived naturally, probably indicating an increase in attachment after concerns about miscarriage were lifted. Similarly, the results of a review study showed that regardless of pregnancy stage, in women who became pregnant following ART, the level of MFA was either similar to or higher than the MFA in women who conceived without ART and they did not require any further attachment interventions (7). It has been shown that these women, despite having more medical complications during pregnancy, such as the need for hospitalization, preterm birth, or cesarean delivery, may have better psychological well-being compared to women who conceived naturally because there is growing hope that they will have a child, which increases their purpose in life (24, 25). Although assisted pregnancy is considered a medicalized pregnancy in most countries, it seems that the possible difficulty in the development of attachment in the first trimester can be resolved as the pregnancy progresses.

In the women who conceived through ART, some MFA sub-scales including “role-taking” and “differentiating of self from fetus” at gestational wk 12 and the sub-scale “attributing characteristics to the fetus” at wk 36 were significantly different than in women who conceived naturally. Delay in emotional attachment to the fetus in the ART women suggests that increased medical interventions in infertility may have adverse consequences on forming a confident maternal identity (18). Although these women feel well supported, the opportunity to engage in a realistic appraisal of the demands and the delights of motherhood, including permission to complain and express uncertainties, can be useful in improving optimal adjustment (26).

Consistent with perinatal attachment theory, which suggests that “the fetus becomes more human to the woman as pregnancy progresses, and eventually the fetus becomes loved both as an extension of self and as an independent object” (27), the results of the present study showed that maternal attachment to the fetus was a developmental process increasing with the progress of the pregnancy. The attachment was significantly higher in both groups at 36 wk of gestation than at wk 12. In the ART group, all the dimensions of MFA increased with pregnancy progression from gestational wk 12 to wk 36 (except “attributing characteristics to the fetus”, which remained unchanged). Similarly, other studies have also shown that attachment improves with increasing gestational age (19, 26, 28). The intensity and frequency of MFA may increase as pregnancy advances because gradually the fetus turns into an independent person, and women envisage their fetus, and a fictionalized image of the appearance and personality of the baby is shaped in their minds (29). In addition, the process of fetus acceptance may develop when the mother first hears the sound of the fetal heart, feels fetal movements or views the image of the fetus in an ultrasound (30).

### PRA

The results showed that PRA in both groups decreased with the progress of the pregnancy, which could be due to reduced concerns about pregnancy loss and the satisfactory results of prenatal screening tests. Contrary to our assumption, there was no significant difference between the two groups in terms of PRA at gestational wk 12 or 36. It seems that over-medicalized pregnancy in pronatalist Iran is associated with high levels of anxiety and stress for all women (both women who conceived by ART and naturally), which may explain why no difference was found in PRA levels between the two groups. The duration of infertility and the history of treatment failure are important factors that may influence the level of women's anxiety in pregnancy (12). However, a greater amount of distress is a recognized feature in ART pregnancies. The psychological effects of infertility treatments on individuals vary and some couples may be more anxious during pregnancy and therefore, psychosocial counseling should be considered as necessary (31). Although general anxiety and PRA affect each other over time (32), we still need to distinguish between general anxiety and PRA. In this regard, the results of previous studies are highly contradictory. Some studies have shown that women who conceived through ART do not experience more PRA than those who conceived naturally (28, 33, 34). In contrast, other studies have reported a greater PRA in pregnant women who conceived through ART (6, 35, 36). The results of a systematic review also revealed that, compared to women who conceived naturally, ART women had greater PRA. Further research is needed on PRA in ART mothers because various studies have used different tools and have been conducted in different settings, and therefore results in this area are inconsistent.

### MFA and PRA

There was an inverse relationship between MFA and PRA only at the 12 ${}^{\text{th}}$ wk of gestation in the women who conceived by ART. This means that increasing the PRA may decrease the MFA at the 12 ${}^{\text{th}}$ wk of gestation. The results of studies about the correlation of PRA with prenatal attachment are contradictory. Some of the studies have shown that attachment is not related to anxiety (26, 34) while other studies have demonstrated that anxiety negatively impacts prenatal attachment (19, 28). According to a recent systematic review, there is a scientific gap in understanding the association between maternal mental health and MFA which limits the theoretical understanding of the MFA construct (37). It seems that there is a positive relationship between mood and MFA with positive health practices (38). Sometimes the development of MFA may be difficult due to anxiety about the loss of pregnancy (39). Therefore, PRA or impaired development of MFA should be taken into account in the prenatal visits of women who conceived through ART particularly in the first trimester of pregnancy. Ultrasound examinations and screening in the first trimester can improve the mother's confidence and her attachment to the fetus.

The strengths of this study were the prospective longitudinal assessment of attachment and PRA with the standardized and validated tool. We also measured PRA by a specific tool. Another strength was the homogeneity of the two groups in terms of marital status, age, education, occupation, economic status, and pregnancy history. However, there were a number of limitations that need to be considered. The study samples were recruited from a referral training hospital in the south of Tehran, where people with low socioeconomic status are usually referred to, which may limit the generalizability of the results. We excluded women with mental health disorders based on their medical history; however, it might have been advantageous if we had evaluated the emotional status of participants using standard instruments before starting the study. Considering that one of the important factors influencing MFA is parity, it would have been better to include only primiparous women. These limitations should be considered in future studies.

## 5. Conclusion 

The study confirmed that maternal attachment to the fetus increases with gestational age, and pregnant women who conceived through ART were as attached to their fetus in the third trimester as other women and did not experience more PRA. However, these women might have difficulties in developing attachment in the first months of pregnancy.

These results could be useful for healthcare professionals caring for pregnant women who have conceived through ART to provide more support to these women especially in the first trimester of pregnancy. These women may be assured that the use of ART is not expected to have negative effects on the development of their attachment to the fetus. Although the level of PRA in these women was similar to that of the control group, these women's concerns may be different from women who conceive naturally. Therefore, healthcare professionals should perform a careful assessment of PRA in women who conceive through ART to provide appropriate psychosocial care to address their real concerns about pregnancy and refer the cases of anxiety to specialized counseling as necessary. It is recommended that future research assess PRA in different wk of pregnancy (that span the full pregnancy instead of pregnancy trimesters) to identify high risk times for anxiety. This can help healthcare professionals to provide ART mothers with more specific care and support.

##  Conflict of Interest

The authors declare that they have no competing interest.
